# The Effectiveness of Patient‐Centered Digital Empowerment Programs in Hematological Cancer Care: A Systematic Review and Meta‐Analysis of Randomized Controlled Trials

**DOI:** 10.1111/wvn.70064

**Published:** 2025-07-24

**Authors:** Merve Gozde Sezgin, Hicran Bektas

**Affiliations:** ^1^ Department of Internal Medicine Nursing Akdeniz University Faculty of Nursing Antalya Turkey

**Keywords:** cancer, digital empowerment programs, meta‐analysis, patient education, systematic review

## Abstract

**Background:**

Hematological cancers impair patients' quality of life (QoL) due to prolonged and complex treatments. Digital empowerment programs enhance patient engagement by supporting symptom management and psychosocial well‐being.

**Aims:**

This study was conducted to examine the effects of patient‐centered digital empowerment programs on hematological cancer care.

**Methods:**

As part of this study, a comprehensive search was conducted in nine databases and the gray literature in March 2025. The screening included randomized controlled trials without any time restrictions. This study adhered to the guidelines outlined in the Preferred Reporting Items for Systematic Reviews and Meta‐Analyses checklist. Fixed‐effect and random‐effect models were used in the meta‐analysis. Cochran's *Q* chi‐square test and *I* ^2^ statistic were applied to assess heterogeneity. Data analysis was performed using the Comprehensive Meta‐Analysis (CMA) 3 software.

**Results:**

A total of seven studies were included in this meta‐analysis. Patient‐centered digital empowerment programs were found to have moderate and positive effects on depression (Hedges' *g* = 0.27, *p* < 0.001), distress (Hedges' *g* = 0.28, *p* < 0.001), self‐efficacy, and QoL (Hedges' *g* = 0.22, *p* < 0.001). There was no significant effect on fatigue levels in patients with hematological cancers (*p* = 0.27), suggesting that digital empowerment programs may not be effective in managing fatigue. The results of the sensitivity analysis support the robustness and reliability of the study findings.

**Linking Evidence to Action:**

Digital empowerment programs may serve as a moderately effective tool in improving depression, distress, self‐efficacy, and QoL among patients with hematological cancers. However, they exhibit limited effects on physical symptoms, particularly in fatigue management. Therefore, more comprehensive and multidisciplinary interventions are needed to address the management of physical symptoms effectively. Patient‐centered digital empowerment programs enable early intervention by assisting healthcare professionals in symptom tracking. Digital solutions enhance care processes by improving patient education, psychosocial support, and self‐management skills.

## 1 Introduction

Hematological cancers, which originate from the blood and lymphatic systems, are classified as malignant diseases and are recognized as a major global health concern (Li et al. 2024; Zomerdijk et al. 2023). According to data from the World Health Organization (WHO), hematological cancers account for 6%–7% of all cancers worldwide. In 2022, approximately 1 million new cases were diagnosed, with over 700,000 deaths recorded in the same year (GLOBOCAN 2022). Current projections indicate that these figures may increase by 40% by 2040 (WHO 2021). The treatment of hematological cancers is complex and long‐term, requiring intensive therapeutic protocols such as chemotherapy, radiotherapy, and stem cell transplantation (Husted Nielsen et al. 2022; Moore et al. 2022). The physical and psychosocial burden associated with these treatments significantly impairs patients' quality of life (QoL) (Elkefi et al. 2023; Keane et al. 2025). Among the most prevalent challenges faced by patients with hematological cancer are fatigue, depression, and loss of self‐efficacy, all of which contribute to reduced treatment adherence (van Bruinessen et al. 2016; Van Der Hout et al. 2020).

Physical, cognitive, and psychosocial symptoms significantly impact the QoL in patients with hematological cancers (Zomerdijk et al. 2023). Fatigue is one of the most prevalent symptoms in this patient group, impacting not only physical energy but also cognitive function and emotional well‐being (Kim et al. 2024; Maguire et al. 2021). Depression and anxiety are commonly observed due to disease‐related uncertainty, physical discomfort, and lack of social support, all of which pose significant challenges to treatment adherence (Jiang et al. 2024; Ziegler et al. 2022). Self‐efficacy is a critical factor in determining patients' ability to manage symptoms and actively participate in their healthcare journey (Tuominen et al. 2024). A decline in self‐efficacy makes it difficult for patients to maintain control over their health and increases their need for additional support (Leach et al. 2022; Schuit et al. 2022). Therefore, symptom management should not be limited to physical and psychosocial support but should also incorporate strategies that enhance self‐efficacy (Van Der Hout et al. 2020).

Patient‐centered digital empowerment programs encompass technological and educational interventions designed to enhance patient engagement in the treatment process, support symptom management, and improve psychosocial well‐being (Kim et al. 2024; Ziegler et al. 2022). Digital health solutions include informative and supportive content, web‐based educational platforms, and remote patient monitoring systems, all of which are designed to help patients manage their own health (Tuominen et al. 2024; Urech et al. 2018). In patients with hematological cancers, digital health tools play a critical role in symptom tracking, medication adherence, psychosocial support, and the development of personalized care plans (Elkefi et al. 2023; Thomas et al. 2022). Nurses play a pivotal role in the implementation of patient‐centered digital empowerment programs, acting as primary healthcare providers in patient education, motivation, and psychological support (Beatty et al. 2016; Pekonen et al. 2020). Their integration into digital empowerment initiatives not only streamlines patient monitoring but also enhances communication, enabling more effective and individualized care delivery (Maguire et al. 2021; Schuit et al. 2022). By leveraging their expertise, nurses can detect symptoms at an early stage, collaborate proactively with the healthcare team to ensure timely interventions, and empower patients to engage more effectively with digital health solutions, ultimately improving health outcomes (Kim et al. 2024; Thomas et al. 2022).

In hematological cancer care, no comprehensive meta‐analysis has been identified that examines the effects of patient‐centered digital empowerment programs on depression, fatigue, self‐efficacy, and QoL. This study aims to analyze the impact of these programs in hematological cancer care and to highlight the significance of their integration into healthcare services from a nursing perspective. Further research is needed in this field, particularly studies evaluating these programs from a nursing standpoint, as such insights are expected to make significant contributions to the care processes for patients with hematological cancers.

## 2 Methods

### 2.1 Design

This meta‐analysis was reported in accordance with The Preferred Reporting Items for Systematic Reviews and Meta‐Analyses (PRISMA) statement checklist (Table S1) (Page et al. 2021). The study design was guided by the Cochrane Handbook for Systematic Reviews, Version 6.4 (Higgins et al. 2023). As this study is a meta‐analysis, ethical approval, and informed consent were not required. The study has been registered in the PROSPERO system (CRD420251000998).

### 2.2 Search Strategies

A comprehensive search was conducted by two independent researchers across Cochrane Library, Web of Science, PubMed, EBSCOhost/CINAHL Complete, MEDLINE, ProQuest, Scopus, ScienceDirect, and Springer Link, as well as the gray literature, covering studies published from inception to March 2025. Articles and gray literature were identified using the snowball sampling method, and in addition to database searches, a manual literature search was also performed. No publication year restrictions were applied, and only randomized controlled trials (RCTs) published in English were included in the study. Medical Subject Headings (MeSH) terms were defined for the database searches, and various combinations of the keywords “hematological cancer,” “digital empowerment,” “patient‐centered,” and “clinical trial” were used (Higgins et al. 2023) (Table S2).

The inclusion criteria included the following:

- Population (P): Individuals aged 18 years or older who have been diagnosed with at least one type of hematological cancer and are at any stage of the disease.
- Interventions (I): Interventions involving patient‐centered digital empowerment programs.
- Comparison (C): Comparisons conducted in studies that include depression, fatigue, distress, self‐efficacy, and QoL scores, without any restrictions on measurement tools.
- Outcomes (O): Studies that assess and report the outcomes of depression, fatigue, distress, self‐efficacy, and QoL as primary or secondary endpoints in patients with hematological cancer.
- Study design (S): Only RCTs published in English were included.

The exclusion criteria included the following:

- Studies comparing two different patient‐centered digital empowerment programs.
- Studies evaluating patient‐centered digital empowerment programs without a control group.
- Studies that do not measure or report depression, fatigue, distress, self‐efficacy, or QoL levels.
- Cross‐sectional studies, non‐randomized studies, cohort studies, retrospective case reports, case–control studies, abstracts, prospective studies, protocols, and studies published in languages other than English.

### 2.3 Data Extraction

A data recording file was created using Microsoft Excel by two independent researchers, and a systematic search was conducted in the databases based on the predefined keywords. Following the search, the researchers cross‐checked the identified studies to verify their accuracy. The extracted data were recorded in the Microsoft Excel file under the following categories: author, year, country, age, hematological cancer stage, name of the patient‐centered digital empowerment program, procedure of the patient‐centered digital empowerment program, characteristics of the patient‐centered digital empowerment program, control group, measurements, and outcomes.

### 2.4 Quality Appraisal

The risk of bias assessment for seven studies was conducted by two independent researchers using the Cochrane Risk of Bias Assessment Tool (RoB2). To resolve potential discrepancies between researchers, a re‐evaluation of the data were planned. However, no disagreements occurred during the risk of bias assessment. The evaluation was carried out using RoB2 under five categories: selection bias, conduct bias, detection bias, attrition bias, reporting bias, and other biases.

All included studies were classified into three categories based on the criteria established (Sterne et al. 2019):

- Low risk of bias: Assigned when all domains were determined to have a low risk of bias.
- Some concerns: Assigned when at least one domain raised some concerns, but no domain was classified as having a high risk of bias.
- High risk of bias: Assigned when at least one domain was identified as having a high risk of bias or when multiple domains raised some concerns.

The selection bias analysis classified all studies as low risk (Beatty et al. 2016; Leach et al. 2022; Maguire et al. 2021; Schuit et al. 2022; Urech et al. 2018; van Bruinessen et al. 2016; Van Der Hout et al. 2020). In three studies, detection bias was categorized as some concerns due to insufficient information regarding the blinding of researchers and participants (Beatty et al. 2016; Leach et al. 2022; Urech et al. 2018). All studies were assessed as low risk in terms of appropriate analytical methods and sufficiency of missing data reporting (Beatty et al. 2016; Leach et al. 2022; Maguire et al. 2021; Schuit et al. 2022; Urech et al. 2018; van Bruinessen et al. 2016; Van Der Hout et al. 2020).

**FIGURE 1:**
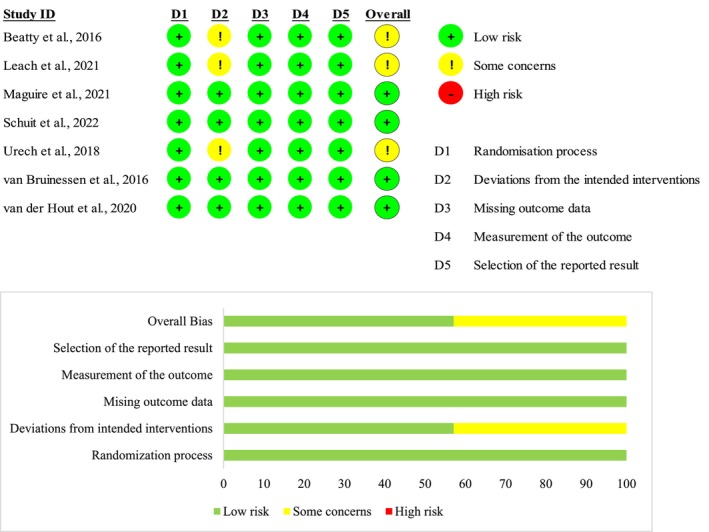
Risk of bias.

In the reporting bias analysis, all studies were classified as low risk, considering the use of appropriate measurement methods and the consistency of reported outcomes (Beatty et al. 2016; Leach et al. 2022; Maguire et al. 2021; Schuit et al. 2022; Urech et al. 2018; van Bruinessen et al. 2016; Van Der Hout et al. 2020). In the overall risk of bias assessment, four studies were categorized as low risk (Maguire et al. 2021; Schuit et al. 2022; van Bruinessen et al. 2016; Van Der Hout et al. 2020), whereas three studies were classified as having “some concerns” regarding overall bias (Beatty et al. 2016; Leach et al. 2022; Urech et al. 2018) (Figure 1).

### 2.5 Data Analysis

A total of seven studies were included in the meta‐analysis (Beatty et al. 2016; Leach et al. 2022; Maguire et al. 2021; Schuit et al. 2022; Urech et al. 2018; van Bruinessen et al. 2016; Van Der Hout et al. 2020). The meta‐analysis incorporated sample sizes, means, standard deviations, *p* values, pre‐post test results, standardized mean differences, and *t*‐test data. Throughout the evaluation process, researchers worked independently to ensure objectivity. The effect size was calculated by dividing the difference between the experimental and control groups by the pooled standard deviation (Borenstein et al. 2014). Due to the presence of small sample groups, Hedges' *g* (Hedges' *g* and 95% confidence interval [CI]) was used instead of Cohen's *d*, as it provides more accurate statistical properties in such cases (Cohen 2013). Effect sizes were classified as large (*d* ≥ 0.80), medium (0.20 < *d* < 0.80), and small (*d* ≤ 0.20) based on standard classification criteria (Higgins et al. 2023). To determine heterogeneity and effect patterns, the *I*‐squared (*I* ^2^) coefficient (0%–40%, 30%–60%, 50%–90%, and 75%–100%) and chi‐squared (*ꭓ* ^2^) tests were used (Deeks et al. 2023). According to Cochran's *Q* statistic and *ꭓ* ^2^ test results, a random‐effects model was applied when *I* ^2^ > 50% and *p* < 0.1 (Higgins et al. 2023). Data analysis was conducted using Comprehensive Meta‐Analysis (CMA) Version 3 and Microsoft Excel software (Borenstein et al. 2014). A mixed‐effects model was used for the subgroup analysis. In this approach, studies within each subgroup were first pooled using a random‐effects model, followed by a fixed‐effects model to test for differences between subgroups (Deeks et al. 2023). Publication bias was assessed using Egger's regression test and Begg's adjusted rank correlation test (Begg and Mazumdar 1994; Deeks et al. 2023; Egger et al. 1997). A two‐tailed *p* value (*p* < 0.05) was considered the threshold for statistical significance.

## 3 Results

### 3.1 Study Selection

**FIGURE 2:**
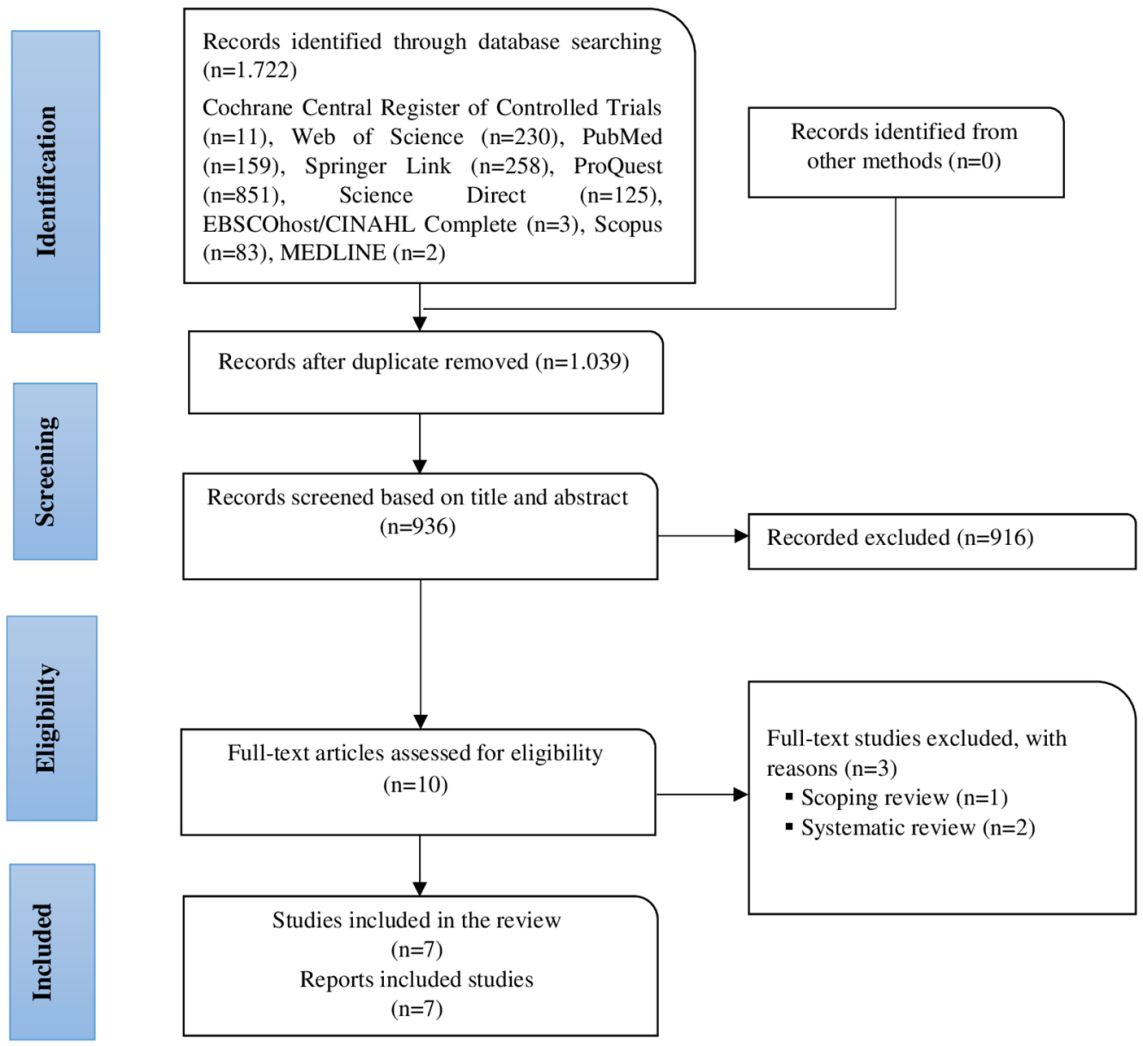
PRISMA flow diagram.

A total of 1722 studies were identified in this meta‐analysis. In the initial screening phase, duplicates were removed using EndNote X8 software (Clarivate Analytics, PA, USA), and 10 studies were selected for full‐text review. However, two studies were excluded for being systematic reviews (Thomas et al. 2022; Tuominen et al. 2024), and one study was excluded for being a scoping review (Kim et al. 2024). As a result, seven RCTs that met the inclusion criteria were selected for analysis. The PRISMA flow diagram is presented in Figure 2.

### 3.2 Characteristics of Patient‐Centered Digital Empowerment Programs

The included studies were conducted between 2016 and 2022. A full list of study characteristics is accessible in Table S3. The sample sizes of the seven RCTs ranged from 30 to 415, with a total of 2044 participants. All reviewed studies included mixed cancer types; however, studies with a high proportion of patients with hematological cancers were prioritized (Beatty et al. 2016; Leach et al. 2022; Maguire et al. 2021; Schuit et al. 2022; Urech et al. 2018; van Bruinessen et al. 2016; Van Der Hout et al. 2020). The mean age of participants in the included studies ranged from 51.57 to 65 years.

The geographical distribution of the included studies shows that three studies were conducted in the Netherlands (Schuit et al. 2022; van Bruinessen et al. 2016; Van Der Hout et al. 2020), 1 in Switzerland (Urech et al. 2018), 1 in Australia (Beatty et al. 2016), 1 in the United States (Leach et al. 2022), and 1 as a multicenter study (Maguire et al. 2021). Regarding cancer stages, four studies did not provide specific information on cancer staging (Beatty et al. 2016; Schuit et al. 2022; Urech et al. 2018; van Bruinessen et al. 2016). One study classified patients into localized, regional, distant, and unknown categories (Leach et al. 2022). Another study categorized patients as stage 0–IV and undefined (Maguire et al. 2021). One study grouped patients into stages I–IV, with an additional category for missing data (Van Der Hout et al. 2020). The digital empowerment programs used in the included studies were diverse. One study utilized Cancer Coping Online (CCO) (Beatty et al. 2016), whereas another implemented Springboard Beyond Cancer and Self‐Management Text Message Program (SBC and self‐management text message program) (Leach et al. 2022). The Advanced Symptom Management System (ASyMS) was used in a multicenter study (Maguire et al. 2021). Two studies employed the Oncokompas program (Schuit et al. 2022; Van Der Hout et al. 2020), one study used Stress‐Aktiv‐Mindern (STREAM) (Urech et al. 2018), and another applied the PatientTIME program (van Bruinessen et al. 2016).

Patient‐centered digital empowerment programs encompass key components such as patient education, providing information and support, offering recommendations, assessing the urgency level of patient care needs, and guiding patients accordingly. CCO is a program that integrates online cognitive‐behavioral therapy applications and supports patient assessments through email and phone reminders (Beatty et al. 2016). SBC provides patients with activity tracking and guidance through a self‐management messaging program (Leach et al. 2022). ASyMS conducts real‐time symptom assessments and provides self‐care recommendations and guidance based on symptom severity (Maguire et al. 2021). Oncokompas offers e‐health self‐management content and informs patients through a three‐tier alert system, categorizing their overall well‐being into mild, moderate, or severe levels, whereas also suggesting self‐care strategies (Schuit et al. 2022). STREAM is a web‐based program incorporating mindfulness‐based stress reduction techniques, recommending patients use eight modules of audio recordings, each lasting between 60 and 90 min, daily (Urech et al. 2018). PatientTIME is a web‐based program designed to enhance patient communication with healthcare providers. It uses short videos to identify patients' communication needs and provide tailored recommendations (van Bruinessen et al. 2016). Additionally, Oncokompas has developed an alert system that sends automated reminders every 3 months, evaluating patients at mild, moderate, or urgent levels while offering self‐care guidance and educational resources (Van Der Hout et al. 2020).

### 3.3 Effects of Patient‐Centered Digital Empowerment Programs

**FIGURE 3:**
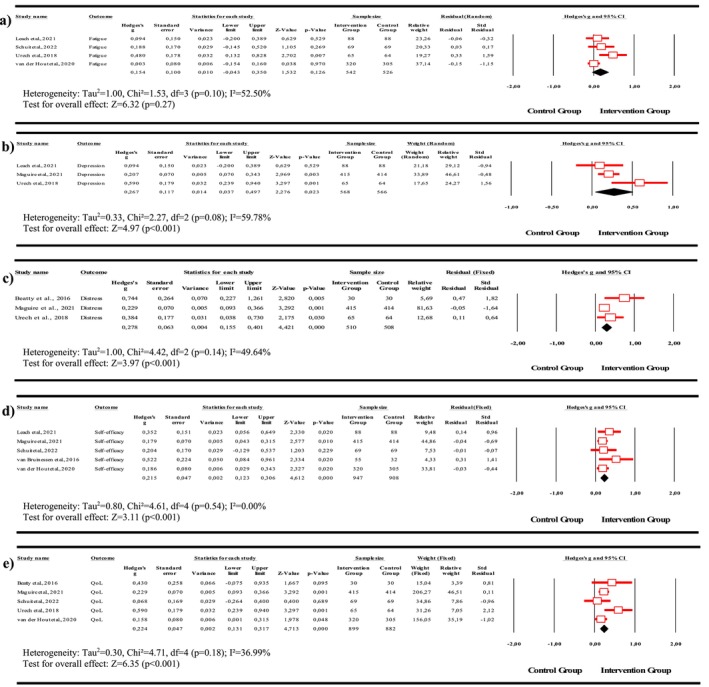
Meta‐analysis results: (a) fatigue, (b) depression, (c) distress, (d) self‐efficacy, and (e) QoL.

This study examined key parameters such as symptoms, self‐efficacy, and QoL (Figure S1). However, studies focusing on sleep disturbances, coping strategies, anxiety, and physical activity were not included in the meta‐analysis, as they did not directly align with the research scope (Figure 3).

#### 3.3.1 Fatigue

Four studies examined the effects of patient‐centered digital empowerment programs on fatigue in patients with hematological cancer (Leach et al. 2022; Schuit et al. 2022; Urech et al. 2018; Van Der Hout et al. 2020). A random‐effects model was used to assess heterogeneity, yielding *p* = 0.10 and *I* ^2^ = 52.50. According to the overall findings, patient‐centered digital empowerment programs did not demonstrate a significant positive effect on fatigue (*p* = 0.27) (Figure 3a). Egger's regression analysis test, Begg's test, and funnel plot results indicated no significant publication bias (*t* = 2.13, *p* = 0.09) (Figure S1).

#### 3.3.2 Depression

Three studies examined the effects of patient‐centered digital empowerment programs on depression in patients with hematological cancer (Leach et al. 2022; Maguire et al. 2021; Urech et al. 2018). A random‐effects model was used to assess heterogeneity, yielding *p* = 0.08 and *I* ^2^ = 59.78. According to the overall findings, patient‐centered digital empowerment programs had a moderate and positive effect on reducing depression levels (Hedges' *g* = 0.27, 95% CI [0.04, 0.50], *p* < 0.001) (Figure 3b). The studies were further analyzed for publication bias using Egger's regression analysis test, Begg's test, and funnel plot data, and no significant publication bias was detected (*t* = 0.53, *p* = 1.00) (Figure S1).

#### 3.3.3 Distress

Three studies examined the effects of patient‐centered digital empowerment programs on distress in patients with hematological cancer (Beatty et al. 2016; Maguire et al. 2021; Urech et al. 2018). A fixed‐effects model was used to assess heterogeneity, yielding *p* < 0.001 and *I* ^2^ = 49.64. According to the overall findings, patient‐centered digital empowerment programs had a moderate and positive effect in reducing distress (Hedges' *g* = 0.28, 95% CI [0.16, 0.40], *p* < 0.001) (Figure 3c). Further analysis using Egger's regression analysis test, Begg's test, and funnel plot data indicated no significant publication bias (*t* = 3.43, *p* = 0.30) (Figure S1).

#### 3.3.4 Self‐Efficacy

Five studies examining the effects of patient‐centered digital empowerment programs on self‐efficacy in patients with hematological cancer were included in this meta‐analysis (Leach et al. 2022; Maguire et al. 2021; Schuit et al. 2022; van Bruinessen et al. 2016; Van Der Hout et al. 2020). The analysis indicated that the overall effect of patient‐centered digital empowerment programs on self‐efficacy was statistically significant (Hedges' *g* = 0.22, 95% CI [0.12, 0.31], *p* < 0.001) (Figure 3d). Since no significant heterogeneity was detected (*p* = 0.54, *I* ^2^ = 0.00), a fixed‐effects model was applied. These findings suggest that patient‐centered digital empowerment programs have a moderate and positive effect on improving self‐efficacy in patients. Further assessment using Egger's regression analysis test, Begg's test, and funnel plot data revealed no significant publication bias (*t* = 2.71, *p* = 0.09) (Figure S1).

#### 3.3.5 Quality of Life

Five studies examining the effects of patient‐centered digital empowerment programs on QoL in patients with hematological cancer were included in this meta‐analysis (Beatty et al. 2016; Maguire et al. 2021; Schuit et al. 2022; Urech et al. 2018; Van Der Hout et al. 2020). To assess heterogeneity, a fixed‐effects model was applied, yielding *p* = 0.18 and *I* ^2^ = 36.99. The findings from the included studies indicate that patient‐centered digital empowerment programs have a statistically significant overall effect on QoL (Hedges' *g* = 0.22, 95% CI [0.13, 0.32], *p* < 0.001) (Figure 3e). Additionally, these programs were found to have a moderate and positive effect on improving self‐efficacy in patients. Further analysis using Egger's regression analysis test, Begg's test, and funnel plot data revealed no significant publication bias (*t* = 0.93, *p* = 0.46) (Figure S1).

### 3.4 Sensitivity Analysis

To enhance the quality of this systematic review and meta‐analysis, a sensitivity analysis was conducted. The sensitivity analysis findings were obtained by systematically removing each included study from the pooled effect sizes. During the sensitivity analysis, each study was excluded one by one, and the effect sizes of all subgroups were recalculated using both random‐effects and fixed‐effects models. The results of the sensitivity analysis confirmed that all included studies had an impact on fatigue, depression, distress, self‐efficacy, and QoL.

## 4 Discussion

The effects of patient‐centered digital empowerment programs on patients with hematological cancer are being increasingly investigated, with evidence suggesting that these programs contribute to symptom management, psychosocial well‐being, and overall QoL (Maguire et al. 2021; Van Der Hout et al. 2020). This meta‐analysis found that digital empowerment programs have moderate positive effects on depression, distress, and self‐efficacy, whereas no statistically significant improvement was observed in fatigue management. These findings highlight digital health applications as a valuable tool for symptom management and psychosocial support, whereas also emphasizing the need for more comprehensive and multidisciplinary approaches in the management of physical symptoms (Beatty et al. 2016; Leach et al. 2022). Therefore, adapting these programs to patients' individual needs and integrating them into nursing care processes may enhance their overall effectiveness.

The limited effect of digital empowerment programs on fatigue in patients with hematological cancer can be attributed to the complex and multifactorial nature of cancer‐related fatigue. The programs evaluated in the included studies primarily focus on psychological support and do not incorporate direct interventions targeting physical symptom management (Leach et al. 2022; Schuit et al. 2022; Urech et al. 2018; Van Der Hout et al. 2020). Since cancer treatment‐related fatigue is closely linked to biological and metabolic processes, programs that provide only psychosocial support may not be sufficient to effectively address this symptom (Schuit et al. 2022; Van Der Hout et al. 2020). Therefore, future digital health solutions should integrate personalized exercise programs, sleep regulation strategies, and nutritional recommendations to enhance fatigue management in patients with hematological cancer.

Digital empowerment programs are more effective in managing depression. By facilitating access to psychosocial support mechanisms, these programs significantly reduce depression levels through psychoeducational content, guidance services, and online support groups (Leach et al. 2022; Maguire et al. 2021; Urech et al. 2018). However, depression is influenced by individual factors, lack of social support, and disease progression, making it challenging to develop a one‐size‐fits‐all model that provides equal benefits for all patients (Kim et al. 2024; Ziegler et al. 2022). Therefore, future digital health solutions should incorporate AI‐driven personalized therapy recommendations, individualized cognitive‐behavioral therapy (CBT) modules, and online psychological support systems integrated with healthcare professionals to optimize depression management.

Digital empowerment programs have been found to have moderate positive effects on distress management. In patients with hematological cancer, distress is associated with uncertainty about the disease, treatment side effects, and psychosocial pressures (Beatty et al. 2016; Maguire et al. 2021; Urech et al. 2018). These programs effectively reduce distress levels by providing informational resources, psychoeducation, and stress management strategies. However, it cannot be assumed that these programs provide equal benefits for all patients (Jiang et al. 2024; Keane et al. 2025). Therefore, future programs should offer dynamic content that can be adapted based on the type and severity of distress. Particularly, interventions supported by mindfulness‐based stress reduction techniques and virtual guidance systems are expected to be more effective in personalized distress management.

Patient‐centered digital empowerment programs not only provide psychosocial benefits but also enhance self‐efficacy, enabling patients to manage their healthcare processes more effectively (Leach et al. 2022; Maguire et al. 2021; Schuit et al. 2022; van Bruinessen et al. 2016; Van Der Hout et al. 2020). Self‐efficacy is a critical factor that allows patients to make informed decisions, adhere to treatment, and take an active role in their healthcare journey (Kim et al. 2024; Leach et al. 2022). Digital health solutions facilitate self‐monitoring, helping patients better understand their symptoms and identify when they need professional support (Elkefi et al. 2023; Ziegler et al. 2022). However, to ensure sustainable improvements in self‐efficacy, these programs should be supported with interactive educational modules, personalized patient guides, and smart tracking systems that simplify individual health monitoring.

The effects of patient‐centered digital empowerment programs on the QoL of patients with hematological cancer have been examined, and these programs have been found to yield significant improvements in overall QoL (Beatty et al. 2016; Maguire et al. 2021; Schuit et al. 2022; Urech et al. 2018; Van Der Hout et al. 2020). Interactive tools that facilitate access to information, provide psychosocial support, and encourage patient engagement have contributed substantially to enhancing patients' QoL (Thomas et al. 2022). However, QoL is a multidimensional concept that extends beyond psychosocial well‐being to encompass physical, emotional, and social dimensions (Tuominen et al. 2024). Therefore, future digital empowerment programs should incorporate more comprehensive models that address all aspects of QoL while focusing on patient satisfaction and holistic care approaches.

To assess the reliability of this study, the risk of bias in the included research was examined. Most studies were classified as low risk for selection bias (Maguire et al. 2021; Schuit et al. 2022; van Bruinessen et al. 2016; Van Der Hout et al. 2020). However, some studies were categorized under some concerns due to insufficient blinding procedures and inadequate explanations of data loss (Beatty et al. 2016; Leach et al. 2022; Urech et al. 2018). The lack of detailed reporting on randomization processes and incomplete adherence to the CONSORT flow diagram introduces methodological uncertainties regarding the reliability of these studies (Sterne et al. 2019). Therefore, future research should ensure more comprehensive reporting of randomization procedures, provide clear explanations for missing data, and fully adhere to CONSORT guidelines to strengthen methodological rigor.

Patient‐centered digital empowerment programs support active patient participation in healthcare processes by providing education, information, and guidance (Beatty et al. 2016; van Bruinessen et al. 2016). Various studies have demonstrated that digital approaches such as online cognitive‐behavioral therapy, self‐management messaging systems, real‐time symptom assessment applications, and web‐based mindfulness techniques, play an effective role in patient care (Leach et al. 2022; Maguire et al. 2021). These methods enable patients to manage their symptoms more effectively, improve access to healthcare services, and facilitate the delivery of personalized care (Schuit et al. 2022; Urech et al. 2018). However, to enhance patient adherence and ensure long‐term effectiveness, digital programs should be designed with user‐friendly interfaces and integrated with personalized reminder systems to optimize engagement and sustainability.

### 4.1 Limitations

This meta‐analysis has several limitations. First, the methodological diversity of the included studies and the variability in the content of the digital empowerment programs made direct comparisons of the results challenging. Second, most studies included mixed cancer types, and there was a limited number of studies specifically focusing on patients with hematological cancer, which affects the generalizability of the findings. Third, a significant portion of the studies assessed short‐ and medium‐term effects, providing insufficient data on long‐term patient outcomes. Finally, differences in healthcare systems and the demographic diversity of patient populations further limit the generalizability of the results.

### 4.2 Linking Evidence to Action

- Digital empowerment programs moderately improve depression, distress, self‐efficacy, and QoL in hematological cancer patients.
- However, they do not significantly impact fatigue management, underscoring the need for broader interventions targeting physical symptoms.
- The study highlights the importance of integrating digital health solutions into patient care and the supportive roles of nurses and healthcare professionals in implementation.
- The reasons for data loss will significantly enhance the reliability of future research.

## 5 Conclusions

This study found that patient‐centered digital empowerment programs have positive effects on depression, distress, self‐efficacy, and QoL in patients with hematological cancer but have a limited impact on fatigue management. In clinical practice, these programs have the potential to support symptom management, enhance psychosocial well‐being, and contribute to personalized care processes. The integration of digital health solutions could offer a more accessible and effective support mechanism, particularly for patients with cancer, improving their treatment processes. Future research should focus on evaluating the long‐term effects of digital empowerment programs, developing digital health solutions specific to patients with hematological cancer, and conducting comparative studies across different healthcare systems. Additionally, strengthening methodological standards, providing detailed reports on randomization processes, and clarifying the reasons for data loss will increase the reliability of future studies. In this regard, multidisciplinary approaches and international collaborations will support the integration of digital health solutions into patient care, contributing to the development of new strategies to improve the QoL for patients with hematological cancer.

## Conflicts of Interest

The authors declare no conflicts of interest.

## Supporting information


**Data S1:** wvn70064‐sup‐0001‐supinfo.docx.
