# Supplementary material for: The Effectiveness of Patient‐Centered Digital Empowerment Programs in Hematological Cancer Care: A Systematic Review and Meta‐Analysis of Randomized Controlled Trials
Source: Worldviews Evid Based Nurs. 2025 Jul 24;22(4):e70064. doi: 10.1111/wvn.70064 (PMC12290137; doi:10.1111/wvn.70064)
Supplement: Supplementary file 1 — Data S1: wvn70064‐sup‐0001‐supinfo.docx. [file WVN-22-0-s001.docx]

**SUPPLEMENTARY MATERIAL TABLE S1** PRISMA Checklist.

| **Section and Topic** | **Item #** | **Checklist item** | **Location where item is reported** |
| --- | --- | --- | --- |
| **TITLE** | | |  |
| Title | 1 | Identify the report as a systematic review. | Title/Abstract |
| **ABSTRACT** | | |  |
| Abstract | 2 | See the PRISMA 2020 for Abstracts checklist. | Page 4/Suppl 1 |
| **INTRODUCTION** | | |  |
| Rationale | 3 | Describe the rationale for the review in the context of existing knowledge. | Page 3-4 |
| Objectives | 4 | Provide an explicit statement of the objective(s) or question(s) the review addresses. | Page 4 |
| **METHODS** | | |  |
| Eligibility criteria | 5 | Specify the inclusion and exclusion criteria for the review and how studies were grouped for the syntheses. | Page 4-5 |
| Information sources | 6 | Specify all databases, registers, websites, organisations, reference lists and other sources searched or consulted to identify studies. Specify the date when each source was last searched or consulted. | Page 4 |
| Search strategy | 7 | Present the full search strategies for all databases, registers and websites, including any filters and limits used. | Suppl 2/Page 4 |
| Selection process | 8 | Specify the methods used to decide whether a study met the inclusion criteria of the review, including how many reviewers screened each record and each report retrieved, whether they worked independently, and if applicable, details of automation tools used in the process. | Figure 1 |
| Data collection process | 9 | Specify the methods used to collect data from reports, including how many reviewers collected data from each report, whether they worked independently, any processes for obtaining or confirming data from study investigators, and if applicable, details of automation tools used in the process. | Page 4-5/Figure 1 |
| Data items | 10a | List and define all outcomes for which data were sought. Specify whether all results that were compatible with each outcome domain in each study were sought (e.g. for all measures, time points, analyses), and if not, the methods used to decide which results to collect. | Page 5 |
|  | 10b | List and define all other variables for which data were sought (e.g. participant and intervention characteristics, funding sources). Describe any assumptions made about any missing or unclear information. | Page 6 |
| Study risk of bias assessment | 11 | Specify the methods used to assess risk of bias in the included studies, including details of the tool(s) used, how many reviewers assessed each study and whether they worked independently, and if applicable, details of automation tools used in the process. | Page 5-6 |
| Effect measures | 12 | Specify for each outcome the effect measure(s) (e.g. risk ratio, mean difference) used in the synthesis or presentation of results. | Page 6 |
| Synthesis methods | 13a | Describe the processes used to decide which studies were eligible for each synthesis (e.g. tabulating the study intervention characteristics and comparing against the planned groups for each synthesis (item #5)). | Page 5-6/Table 1 |
|  | 13b | Describe any methods required to prepare the data for presentation or synthesis, such as handling of missing summary statistics, or data conversions. | Page 5-6 |
|  | 13c | Describe any methods used to tabulate or visually display results of individual studies and syntheses. | Page 5-6 |
|  | 13d | Describe any methods used to synthesize results and provide a rationale for the choice(s). If meta-analysis was performed, describe the model(s), method(s) to identify the presence and extent of statistical heterogeneity, and software package(s) used. | Page 5-6 |
|  | 13e | Describe any methods used to explore possible causes of heterogeneity among study results (e.g. subgroup analysis, meta-regression). | Page 5-6 |
|  | 13f | Describe any sensitivity analyses conducted to assess robustness of the synthesized results. | Page 5-6 |
| Reporting bias assessment | 14 | Describe any methods used to assess risk of bias due to missing results in a synthesis (arising from reporting biases). | Page 5-6 |
| Certainty assessment | 15 | Describe any methods used to assess certainty (or confidence) in the body of evidence for an outcome. | Page 5-6 |
| **RESULTS** | | |  |
| Study selection | 16a | Describe the results of the search and selection process, from the number of records identified in the search to the number of studies included in the review, ideally using a flow diagram. | Page 6-7/Figure 1 |
|  | 16b | Cite studies that might appear to meet the inclusion criteria, but which were excluded, and explain why they were excluded. | Page 5 |
| Study characteristics | 17 | Cite each included study and present its characteristics. | Page 7 |
| Risk of bias in studies | 18 | Present assessments of risk of bias for each included study. | Page 5 |
| Results of individual studies | 19 | For all outcomes, present, for each study: (a) summary statistics for each group (where appropriate) and (b) an effect estimate and its precision (e.g. confidence/credible interval), ideally using structured tables or plots. | Figure 2/Page 7-8 |
| Results of syntheses | 20a | For each synthesis, briefly summarise the characteristics and risk of bias among contributing studies. | Page 4 |
|  | 20b | Present results of all statistical syntheses conducted. If meta-analysis was done, present for each the summary estimate and its precision (e.g. confidence/credible interval) and measures of statistical heterogeneity. If comparing groups, describe the direction of the effect. | Figure 2/Page 8-10 |
|  | 20c | Present results of all investigations of possible causes of heterogeneity among study results. | Page 8-9 |
|  | 20d | Present results of all sensitivity analyses conducted to assess the robustness of the synthesized results. | Page 9 |
| Reporting biases | 21 | Present assessments of risk of bias due to missing results (arising from reporting biases) for each synthesis assessed. | Page 5-6 |
| Certainty of evidence | 22 | Present assessments of certainty (or confidence) in the body of evidence for each outcome assessed. | Page 8-11 |
| **DISCUSSION** | | |  |
| Discussion | 23a | Provide a general interpretation of the results in the context of other evidence. | Page 11-12 |
|  | 23b | Discuss any limitations of the evidence included in the review. | Page 11-13 |
|  | 23c | Discuss any limitations of the review processes used. | Page 12-13 |
|  | 23d | Discuss implications of the results for practice, policy, and future research. | Page 13-14 |
| **OTHER INFORMATION** | | |  |
| Registration and protocol | 24a | Provide registration information for the review, including register name and registration number, or state that the review was not registered. | Page 4 |
|  | 24b | Indicate where the review protocol can be accessed, or state that a protocol was not prepared. | Page 4 |
|  | 24c | Describe and explain any amendments to information provided at registration or in the protocol. | Page 4 |
| Support | 25 | Describe sources of financial or non-financial support for the review, and the role of the funders or sponsors in the review. | Title Page |
| Competing interests | 26 | Declare any competing interests of review authors. | Title Page |
| Availability of data, code and other materials | 27 | Report which of the following are publicly available and where they can be found: template data collection forms; data extracted from included studies; data used for all analyses; analytic code; any other materials used in the review. | Title Page |

*From:*  Page MJ, McKenzie JE, Bossuyt PM, Boutron I, Hoffmann TC, Mulrow CD, et al. The PRISMA 2020 statement: an updated guideline for reporting systematic reviews. BMJ 2021;372:n71. doi: 10.1136/bmj.n71

For more information, visit: <http://www.prisma-statement.org/>

**SUPPLEMENTARY MATERIAL TABLE S2** Search strategy.

| Cochrane Central Register of Controlled Trials | cancer OR hematologic malignancy OR hematologic OR hematologic cancer OR hematology OR hematological in All Text AND empowerment OR self-management OR self-car* OR self-manag* OR self-care OR empower* OR coping* OR perceived control* OR activation OR action* OR self-efficac* in All Text AND digital* OR digitalisation* OR digitalization* OR e-health OR ehealth OR mhealth* OR electronic health* OR telecare* OR mobile health OR digitisation* OR mobile-based* OR tele-based* OR digital technology in All Text AND randomized controlled trial OR all random OR RCT OR control OR clinical trial OR clinical trials OR controlled study OR evidence based OR best practice OR evidence synthesis OR experiment OR trial OR questionnaire OR survey OR follow up OR interview OR focus group OR experience OR experiences OR observation OR mixed method in All Text - (Word variations have been searched) |
| --- | --- |
| PubMed | ( cancer OR hematologic malignancy OR hematologic OR hematologic cancer OR hematology OR hematological ) AND ( empowerment OR self-management OR self-car* OR self-manag* OR self-care OR empower* OR coping* OR perceived control* OR activation OR action* OR self-efficac* in All Text AND digital* OR digitalisation* OR digitalization* OR e-health OR ehealth OR mhealth* OR electronic health* OR telecare* OR mobile health OR digitisation* OR mobile-based* OR tele-based* OR digital technology ) AND Filters: clinical trial, clinical study, multicenter study, controlled clinical trial, randomized controlled trial |
| Springer Link | cancer OR hematology AND empowerment AND digital AND randomized controlled trial |
| ProQuest | ab( cancer OR hematologic malignancy OR hematologic OR hematologic cancer OR hematology OR hematological ) AND ab( empowerment OR self-management OR self-car* OR self-manag* OR self-care OR empower* OR coping* OR perceived control* OR activation OR action* OR self-efficac* in All Text AND digital* OR digitalisation* OR digitalization* OR e-health OR ehealth OR mhealth* OR electronic health* OR telecare* OR mobile health OR digitisation* OR mobile-based* OR tele-based* OR digital technology) AND ab( randomized controlled trial OR all random OR RCT OR control OR clinical trial OR clinical trials OR controlled study OR evidence based OR best practice OR evidence synthesis OR experiment OR trial OR questionnaire OR survey OR follow up OR interview OR focus group OR experience OR experiences OR observation OR mixed method) |
| Science Direct | ("cancer" OR "hematology") AND ("empowermen") AND ("digital") AND ("randomized controlled trial") Filtered Research articles |
| EBSCOhost/CINAHL Complete | TX ( cancer OR hematologic malignancy OR hematologic OR hematologic cancer OR hematology OR hematological ) AND ( empowerment OR self-management OR self-car* OR self-manag* OR self-care OR empower* OR coping* OR perceived control* OR activation OR action* OR self-efficac* ) in All Text AND ( digital* OR digitalisation* OR digitalization* OR e-health OR ehealth OR mhealth* OR electronic health* OR telecare* OR mobile health OR digitisation* OR mobile-based* OR tele-based* OR digital technology ) AND ( randomized controlled trial OR all random OR RCT OR control OR clinical trial OR clinical trials OR controlled study OR evidence based OR best practice OR evidence synthesis OR experiment OR trial OR questionnaire OR survey OR follow up OR interview OR focus group OR experience OR experiences OR Observation OR mixed method ) Full-text " |
| Scopus | TITLE-ABS-KEY ( "cancer" OR "hematologic malignancy" OR "hematologic" OR "hematologic cancer" OR "hematology" OR "hematological" ) AND TITLE-ABS-KEY ( "empowerment" OR "self-management" OR "self-car*" OR "self-manag*" OR "self-care" OR "empower*" OR "coping*" OR "perceived control*" OR "activation" OR "action*" OR "self-efficac*" ) AND TITLE-ABS-KEY ( "digital*" OR "digitalisation*" OR "digitalization*" OR "e-health" OR "ehealth" OR "mhealth*" OR "electronic health*" OR "telecare*" OR "mobile health" OR "digitisation*" OR "mobile-based*" OR "tele-based*" OR "digital technology" ) AND TITLE-ABS-KEY ( "randomized controlled trial" OR "all random" OR "RCT" OR "clinical trial" OR "clinical trials" OR "controlled study" OR "evidence based" ) |
| MEDLINE | TX ( cancer OR hematologic malignancy OR hematologic OR hematologic cancer OR hematology OR hematological ) AND ( empowerment OR self-management OR self-car* OR self-manag* OR self-care OR empower* OR coping* OR perceived control* OR activation OR action* OR self-efficac* ) in All Text AND ( digital* OR digitalisation* OR digitalization* OR e-health OR ehealth OR mhealth* OR electronic health* OR telecare* OR mobile health OR digitisation* OR mobile-based* OR tele-based* OR digital technology ) AND ( randomized controlled trial OR all random OR RCT OR control OR clinical trial OR clinical trials OR controlled study OR evidence based OR best practice OR evidence synthesis OR experiment OR trial OR questionnaire OR survey OR follow up OR interview OR focus group OR experience OR experiences OR Observation OR mixed method ) Full-text " |
| Web of Science | 5# #4 AND #3 AND #2 AND #1  T1=(cancer OR hematologic malignancy OR hematologic OR hematologic cancer OR hematology OR hematological)  T2=(empowerment OR self-management OR self-car* OR self-manag* OR self-care OR empower* OR coping* OR perceived control* OR activation OR action* OR self-efficac* )  T3=(digital* OR digitalisation* OR digitalization* OR e-health OR ehealth OR mhealth* OR electronic health* OR telecare* OR mobile health OR digitisation* OR mobile-based* OR tele-based* OR digital technology )  T4=(randomized controlled trial OR all random OR RCT OR control OR clinical trial OR clinical trials OR controlled study OR evidence based OR best practice OR evidence synthesis OR experiment OR trial OR questionnaire OR survey OR follow up OR interview OR focus group OR experience OR experiences OR Observation OR mixed method ) |

**Supplementary Material Table S3** The characteristics of included studies.

| Author, Year/ Country | Participants | | | | | | | | |  | |  | | Intervention | | | Outcome | Results |
| --- | --- | --- | --- | --- | --- | --- | --- | --- | --- | --- | --- | --- | --- | --- | --- | --- | --- | --- |
|  | **Patients with cancer** | **Age** | | **Cancer stage** | **Sample size** | | | | |  | **Experimental** | | | |  | **Control** | **Measurement** |  |
|  |  | **experimental*** | **control*** |  | **experimental*** | | | | **control*** | **Collaborative competences** | **The name of digital empowerment** | | **The procedure of digital empowerment** | | **Features of digital empowerment programs** |  |  |  |
| Beatty et al., 2016, Australia | Lymphoma and other cancers | Mean (SD)  51.57±10.10 | Mean (SD)  53.90±9.48 | No  information | | 30 | | | 30 | Quality of life  Distress | CCO* | | CCO is a program consisting of three main components: cognitive-behavioral therapy (CBT)-based activities such as psychoeducation, worksheets, quizzes, and relaxation and meditation exercises, as well as written survivor references and citations. Additionally, CCO offers the opportunity for personal journaling/blogging and connecting with websites. | | - After patients reviewed the three main sections, they were automatically directed to a post-treatment assessment. Patients who completed the guidance process received email reminders in the 1st and 2nd weeks, and a one-time phone reminder was used for patients who did not respond by the end of the 3rd week. Following this procedure, assessments were conducted at 3 months and 6 months. | The web-based attention control | - EORTC QLQ-C30** - PSS-SR** | - Quality of life: p=0.097 - Distress: p=0.005 |
| Leach et al., 2021, USA | Blood/Lymph and other cancers | N (%)  Male:9 (10.2)  Female:78 (88.6) | N (%)  Male: 7 (8.0)  Female: 81 (92.0) | Localised, regional, distant, and unknown | | | 88 | | 88 | Self-efficacy  Fatigue  Depression | SBC and self-management text message program* | | An updated SBC program was introduced, which includes a login feature for activity tracking. Additionally, patients were provided with a text message service where they could receive responses to questions related to their condition. | | - The text message service includes a 4-week self-management program. The text message program lasted for 28 days starting from the registration date, with patients receiving 1 to 5 messages daily. | Access to a website built for the study | - SEMCD-S** | - Self-efficacy: p=0.02 - Fatigue: p=0.23 - Depression: p=0.53 |
| Maguire et al., 2021, Austria, Greece, Norway, Ireland, and UK | Hodgkin’s or Non-Hodgkin’s Lymphoma and other cancers | Mean (SD)  51.9±12.4 | Mean (SD)  52.9±12.1 | Stages 0-IV and undefined | | | 415 | 414 | | Depression  Self-efficacy  Quality of Life  Distress | ASyMS* | | ASyMS enabled the real-time monitoring and management of chemotherapy-induced toxicities 24 hours a day. Patients were directed to complete surveys via ASyMS that assess 10 symptoms and allow for self-reporting. Additionally, patients benefited from personalized self-care recommendations provided through ASyMS, available 24/7. | | - Two alert levels were created for symptoms (mild-moderate and urgent). The response time was set to 8 hours for mild-moderate symptoms and 30 minutes for urgent situations. This procedure was followed throughout 6 chemotherapy cycles. | Standard care | - STAI-R** - CASE-Cancer** - FACT-G** - MSAS** | - Depression: p=0.003 - Self-efficacy: p=0.01 - Quality of Life: p<0.001 - Distress: p<0.001 |
| Schuit et al., 2022, Netherlands | Hematological cancer and other cancers | Mean (SD)  60.0±12.7 | Mean (SD)  62.3±11.9 | No information | | | 69 | 69 | | Self-efficacy  Quality of Life  Fatigue | Oncokompas | | Oncokompas is an eHealth self-management program consisting of three steps: Measure, Learn, and Act. In the Measure step, a survey was conducted to identify appropriate topics for the patients. In the Learn step, information and feedback were provided about results tailored to the patients' health status, personal characteristics, and preferences. In the Act step, patients were given a personalized overview of supportive care options, with professional guidance options recommended when necessary. | | - A three-tiered alert system was created for overall well-being (mild-moderate-severe). - Mild level indicated that the patient was generally well; the moderate level highlighted the need for attention and support; and the severe level emphasized the patient's need for immediate care and support, along with self-care recommendations provided accordingly. | Standard care | - GSE** - EORTC-QLQ-C15-PAL** | - Self-efficacy: p=0.23 - Quality of Life: p=0.69 - Fatigue: p=0.27 |
| Urech et al., 2018, Switzerland | Lymphoma and other cancers | Median age, years  51(46-57) | Median age, years  53(46-58) | No information | | | 65 | 64 | | Depression  Quality of Life  Fatigue  Distress | STREAM* | | The web-based STREAM program is a program that incorporates cognitive-behavioral therapy and mindfulness-based stress reduction techniques. The program consists of eight modules, each of which can be completed in 60 to 90 minutes. Additionally, the program encourages the daily use of downloadable audio files that include relaxation and guided imagery exercises. | | - Patients were asked to complete one module each week. Additionally, written feedback was provided to them weekly via email. | Standard care | - HADS** - FACIT-F** | - Depression: p=0.001 - Quality of Life: p=0.001 - Fatigue: p=0.007 - Distress: p=0.03 |
| van Bruinessen et al., 2016, Netherlands | Hodgkin’s or Non-Hodgkin’s Lymphoma and other cancers | Mean (SD)  55±13 | Mean (SD)  56±14 | No information | | | 55 | 32 | | Self-efficacy | PatientTIME* | | The web-based PatientTIME program is designed to support patients in gaining more control over their communication with their healthcare team. The program consists of 58 short video clips featuring simulated patients with various communication skills. The selected video content from the video library was based on the communication preferences and needs that patients reported prior to their visits. | | - The duration of the short videos ranged from 47 to 180 seconds on average. Patients received two reminders as part of the program. | Standard care | - PEPPI** | - Self-efficacy: p=0.02 |
| van der Hout et al., 2020, Netherlands | Hodgkin’s or Non-Hodgkin’s Lymphoma and other cancers | Median age, years  65(56-71) | Median age, years  65(57-71) | Stages I-IV and missing | | | 320 | 305 | | Self-efficacy  Quality of Life  Fatigue | Oncokompas | | The web-based eHealth application Oncokompas is a program designed to monitor general cancer symptoms and tumor-specific symptoms, assess quality of life, provide feedback and information on scores, and offer a personalized overview of supportive care options. Oncokompas consists of three steps: Measure, Learn, and Act. | | - To encourage the use of Oncokompas, automated reminders were sent every three months. Additionally, a help desk service was provided, allowing patients to contact support via email or phone. - A three-tiered alert system (mild-moderate-urgent) was implemented in the program. Mild level indicated that there was no high risk to well-being; moderate level signified a high risk to well-being; and urgent level indicated a seriously high risk to well-being. Patients who scored in the moderate or urgent levels received feedback on their issues and personalized self-care recommendations. - Evaluations were conducted one week after the intervention, as well as during follow-up processes at the 3rd and 6th months. | Standard care | - GSE** - EORTC QLQ-NHL-HG29** | - Self-efficacy: p=0.02 - Quality of Life: p=0.048 - Fatigue: p=0.97 |

*Cancer Coping Online: CCO; SBC and self-management text message program: Springboard Beyond Cancer and Self-Management Text Message Program; Advanced Symptom Management System: ASyMS; Stress-Aktiv-Mindern: STREAM

**Functional Assessment of Cancer Therapy—General: FACT-G; the Stanford Self-Efficacy for Managing Chronic Disease Scale: SEMCD-S; Communication and Attitudinal Self-Efficacy scale for cancer: CASE-Cancer; State-Trait Anxiety Inventory—Revised: STAI-R; the General Self−Efficacy scale: GSE; Perceived Efficacy in Patient-Physician Interactions: PEPPI; European Organisation for Research and Treatment of Cancer Quality of Life Questionnaire Non-Hodgkin lymphoma: EORTC QLQ-NHL-HG29; Hospital Anxiety and Depression Scale: HADS; Functional Assessment of Chronic Illness Therapy–Fatigue: FACIT-F; the Posttraumatic Stress Scale—Self-Report: PSS-SR

**SUPPLEMENTARY MATERIAL FIGURE S1** Funnel plots.

Fatigue

Depression

Distress

Self-efficacy

QoL
